# Supplementary material for: Community led testing among people who inject drugs: A community centered model to find new cases of HIV and Hepatitis C in Nepal
Source: PLoS One. 2021 May 28;16(5):e0252490. doi: 10.1371/journal.pone.0252490 (PMC8162586; doi:10.1371/journal.pone.0252490)
Supplement: Attachment — Submitted filename: Response sheet-updated.docx [file pone.0252490.s001.docx]

Dear Editor and Reviewers,

Thank you very much for reviewing our manuscript and providing critical feedback to improve the manuscript. We are addressing your comments and feedback and revised the manuscript accordingly. Please see the responses made in the below tables.

**Table 1 Response to the editor’s comments**

| **Comments** | **Responses** |
| --- | --- |
| 1. Please ensure that your manuscript meets PLOS ONE's style requirements, including those for file naming. The PLOS ONE style templates can be found at | We followed the PLOS ONE's style requirements as advised. |
| 2. Please review your reference list to ensure that it is complete and correct. If you have cited papers that have been retracted, please include the rationale for doing so in the manuscript text, or remove these references and replace them with relevant current references. Any changes to the reference list should be mentioned in the rebuttal letter that accompanies your revised manuscript. If you need to cite a retracted article, indicate the article’s retracted status in the References list and also include a citation and full reference for the retraction notice. | We have added the references as advised by the reviewers. We mentioned in the respective comments for the required citation. |
| 3. Please consider including more information on the number as per the of interviewers, their training and characteristics; and please provide the interview guide used. | Two qualitative researchers received 3 days training covering using topic guides, selecting participants, maintaining ethical procedures, recording and transcribing the information. We mentioned detail in the text.  We revised the text please see page#6, lines #151-159 |
| 4. We note that you have indicated that data from this study are available upon request. PLOS only allows data to be available upon request if there are legal or ethical restrictions on sharing data publicly. For information on unacceptable data access restrictions, please see http://journals.plos.org/plosone/s/data-availability#loc-unacceptable-data-access-restrictions. | We will made available the data through correspondence with the request of needy people. We may not be able to share the data publicly due to ethical reason of confidentiality of participants as the study is confined in the particular districts with covering particular organizations. Also, there are several information related to the participants who tested positive for HIV and HCV such as linkage to care, their past injecting behavior and their qualitative response where they experience in the testing such as police interference etc.  Please correspond to   1. National Center for AIDS and STDs Control (Government body of Nepal) will be responsible for providing the data.   Email: [ncasc@ncasc.gov.np](mailto:ncasc@ncasc.gov.np)   2. National Association of People Living with HIV/AIDS in Nepal   info@napn.org.np |
| 5. Thank you for stating the following in the Acknowledgments Section of your manuscript:  [Further, we would like to acknowledge the support received from staffs of Save the children, NCASC, NAP+N, and NPHL.]  We note that you have provided funding information that is not currently declared in your Funding Statement. However, funding information should not appear in the Acknowledgments section or other areas of your manuscript. We will only publish funding information present in the Funding Statement section of the online submission form.  Please remove any funding-related text from the manuscript and let us know how you would like to update your Funding Statement. Currently, your Funding Statement reads as follows:  [The author(s) received no specific funding for this work.]  Please include your amended statements within your cover letter; we will change the online submission form on your behalf. | We omitted the financial related information in the acknowledgment.  We mentioned the funding related information in the funding information.  We also mentioned it in the cover letter with amended statements  Please see the page #16, line#460  We will mention financial related information in the relevant section |
| 6. Please amend your list of authors on the manuscript to ensure that each author is linked to an affiliation. We note that affiliation 3 is not linked. Authors’ affiliations should reflect the institution where the work was done (if authors moved subsequently, you can also list the new affiliation stating “Current affiliation stating “Current affiliation…” as necessary). | We amended the list of authors and linked the author with affiliation 3 |
| 7. Please ensure that you refer to Figure 1 in your text as, if accepted, production will need this reference to link the reader to the figure. | We mention Figure 1 in line #193, page #7 |

**Table 2 : Responses to the reviewer’s comments**

| Comments | Responses |
| --- | --- |
| **Reviewer 1** |  |
| The manuscript describes an important contribution to the literature in Nepal - the role of peer led approaches to addressing HIV and HCV testing. While these data have been repeatedly shown in many other studies, it is important to show this is feasible in Nepal. Overall, the manuscript is well-written. However, there is lack of sufficient detains on the peer-led approach itself. In particular, there is a need for clarification of characteristics of IRWs, who are defined as having “similar backgrounds to PWID”. What characteristics are included to determine this similarity? Current/former PWIDs themselves? Similar age? The definition of peers needs more clarification in order to determine potentially what components might important to recommend for future interventions. | Thank you for your comment. Yes, we agree that the purpose of the study is to show that the study is feasible and of importance in Nepal.  We have provided detail in peer led approach as advised. We described the characteristics of IRWs such as those who used drugs from the same areas, they are former IRWs but currently not injecting and associated with the NGO as peer educators. Peer means those who have the similar injecting background in the past and currently working as paid in CSOs working in HIV prevention programs.  Please see Page #4, lines #100-102 |
| Figure 1 –coloring scheme makes it difficult to read. There is no text describing/summarizing what is going on in this figure. This would be helpful. | We have described the algorithm in sub-title “biological test” We have revised for this clarity. Please see # page7, #lines 193-195 and #201-206 |
| Define advantaged vs. disadvantaged Janajati | We clarified as advantaged (Newar, Gurung, and Thakali) and disadvantaged Janajati (Other than Newar Gurung, and Thakali) as described by the health management information system  Please see lines #256-257, page#10 |
| Define reused needles—does this include only individuals who resused needles but did not share it? The discussion statement—“resused of needles….may have increased the risk of …transmission” implies that these were also potentially shared? Please clarify as resusing a needle only used by oneself should not pose any transmission risk. | We revised for the clarity  “Reuse of needles by oneself and received from others and needle/ syringe sharing may have increased the risk..”,  Please see lines #425, Page #15 |
| Word choice of “proved” in the second sentence of the discussion—rephrase to “shown to be” or similar. | We revised it as advised  Please see line #389, page #14 |
| **Reviewer 2** |  |
| Importantly, it is unclear why sociodemographic characteristics; risk characteristics; and past testing, prevention, stigma, and current testing are stratified by gender rather than HCV screening status, which is a primary outcome of the analysis. Additional details and recommendations are outlined below. | We stratified the gender because current drug use among women was higher compared to men so that we wanted to show gender stratified sociodemographic characteristics; risk characteristics; and past testing, prevention, stigma, and current testing. However, we did not see the differences in HCV results by gender.  We have shown the descriptive analysis stratified HCV screening results in Table 4 and 5 covering prevention, and risk characteristics. Table # 4 and #5 have been merged as Table #4, Table #6 changed as Table #5 as advised. |
| ABSTRACT - Line 33: “People who inject drugs” does not require capitalization - Line 40: “mixed methods” (rather than mix-method) is the standard terminology for this approach | We revised as suggested  Please see lines #36, page #2 and lines #43. |
| INTRO - Lines 83-85: “In addition, HCV testing services are only available in clinic-based settings, and PWIDs may not feel comfortable to seeking these testing services from the facilities.” – While this is accurate, a citation would strengthen this statement. - Lines 85-87: This statement would be strengthened by noting that these social/environmental challenges are barriers to HIV/HCV testing. | We have provided citation of the argument as advised  Please Reference #12, line #90, page#4 |
| METHODS - Lines 126-127: It is somewhat unclear how the final sample size of 1029 was determined. Was there a calculation to determine how many persons in excess of 980 were needed? Please clarify. | We have not considered factors to increase the sample size from 980 to 1029. We did not calculate the individual factors contribution. However, from our convenience sampling, We also aimed to identify new cases of HIV in the study areas and purposefully increased the sample size to 1029. We have added the justification. Please see lines #135-136, page# 5 |
| Line 129: Typographical error. I believe this should read, “the location of secret places” | We corrected as advised, please see lines #144 page#6 |
| - There is quite a large number of uncommon acronyms in this section, and this makes the methodology a bit hard to follow. I would suggest using PWID, HIV, HCV, and CLT, (Maybe IBBS and IRWs) then spell out any of the remaining acronyms for clarify. | We have elaborated the new acronyms used in the whole text as advised. |
| - Lines 141-142: “Validated IDIs and FGD topic guides were used in the study.” Are citations available for these validated measures? | IDIs and FGDs were validated by the project itself with the experts, government officials, and relevant stakeholders. These are the original topic guides and not the adopted one.  We added clarification as We used topic guides for IDIs and FGDs after conducting validation testing with the government and non-government stakeholders including experts in the study.  Please see lines #160-161, page#6 |
| - Lines 160-163: It is unclear whether these prevention activities were assessed qualitatively or quantitatively. Please specify. | The measure was assessed quantitatively and qualitatively. We added the burden of risk factors as to provide clarity.  Please see lines 191-192, page #7 |
| In the introduction, the authors note that the aims of the study were to “examine the acceptability and feasibility of a CLT model of testing for HIV and HCV among PWIDs and linking PWIDs to the available tratment [note spelling error: “treatment”], care, and support services.” However, the methods section lacks information about how linkage to treatment, care, and support services was measured and assessed. This is particularly unclear given the cross-sectional design of this study. | We have specified as of algorithm. As the identified cases were all referred to the treatment, care and support services.  Please see lines #193-206, page #7 |
| - There is no reference to Figure 1 in the text, though I assume that this should appear in the methods section. | This is described in the algorithm testing. The project itself developed it and followed Government testing algorithm. |
| Please expand the data analysis section to detail which specific descriptive analyses were conducted. | We have added stratified by gender and stratify by the HIV screening result.  We used chi-squared test to see the gender differences in risk characteristics, sociodemographic characteristics, and characteristics according to the HCV-positive and HCV-positive results. We applied t-test for continuous variables.  Please see lines #222-224, page #9 |
| - How were the final variables included in the multivariate analysis determined? Please specify. | We applied univariate analysis and developed model covering age, sex, marital status, education, risk characteristics, prevention characteristics, and rehabilitation characteristics as these are considered the important risk factors for HCV results in Nepal.  Please see lines #226-239, page #9 |
| RESULTS - I would suggest moving Tables 1-3 to a supplemental file. While stratifying these data by gender is informative, stratification by HCV screening status (as is done in tables 4-5) more directly supports the aims of this study. It may also be informative to present some of the risk characteristics and/or past testing, prevention, stigma, etc. by HCV status; I will leave this determination to the authors. | We are agreed with the suggestion. The paper reads about HCV screening.  We also tend to describe the characteristics of the participants by gender. We decided to retain as it not only for HCV screening result. As reviewer suggested it is up to us. |
| - It is unclear why risk-related characteristics and past testing, etc. are stratified and presented by gender. This strikes me as a secondary analysis that would be more appropriate presented/summarized in a supplemental files. The authors may consider presenting a portion of these data stratified by HCV status in one additional primary table. | The purpose of showing descriptive analysis is just to describe the sociodemographic characteristics. Even though our conclusion is not the gender-based results, we have found that more women were current drug users and so on. |
| - Line 226: It is unclear how current vs. past drug use was defined. | Those who are injecting drugs less than 3months were current drug users and those who injected drugs prior to three months are pas drug users.  Please see lines #180-183, page #7 |
| - Risk related characteristics of participants by gender and Past testing, prevention exposure according to gender should be relocated to a supplemental file. It would be more informative to present some of these findings stratified by HCV status, as this is the primary outcome of interest in this study. | We have presented in the table for HCV. We clarified in the previous comment about our flow of study. |
| - Line 286: It is unclear how “started OST” was defined. For example, was this ever, in the past year, or current OST? | Current OST, we revised it as who are on OST  See table #5, page #24 |
| - Sections of the qualitative results should be reviewed for academic tone. For example, lines 340-341: “However, they never got discouraged and kept on going for testing and finding the community.” This sentence should be re-worked for clarity, informal terminology should be removed, and it should be framed based on what IRWs reported in interviews (e.g., “Some IRWs reported…”). | We revised it as advised in the qualitative results section as:  However, the majority of the IRWs reported that this incidence did not affect them in continuation of testing and finding new people for the testing.  Please see lines #362-#366, page#13 |
| - The second and third quotes (page 13) are a bit difficult to follow. Is it possible for these translations to be enhanced? | We revised second and third quote as suggested  See page #14, lines #376-381 |
| DISCUSSION - Line 346: This should read “This study is the first of its kind…” - Lines 359-361: “The lower rate of HIV could be due to better utilization of testing services and their exposure to prevention services.” – Could the authors elaborate here? Are HIV testing services more accessible/available in these regions? | Revised as advised, lines #389  HIV testing services are more accessible in the study areas and have better linkage to treatment and care.  Lines #404, 405, Page #14. |
| TABLES/FIGURES - Figure 1 is difficult to discern; a higher-resolution image of this figure should be provided. - Findings stratified by gender should be relocated to a supplemental file (see details above) - In Table 6, the referent categories for all characteristics should be included. For example, “Needle sharing (yes)” and “Age (per year)”. Educational level should be binary with the referent category defined, unless mean schooling years was used instead. - In the text (and possible in a note below the table) the authors should specify the recall period (e.g., needle sharing in the past year or ever?) and define “OST started” and “attended rehabilitation center” (i.e., are this ever, past year, or current?) - Tables 4 and 5 could be collapsed into a single table. - In Table 6, the authors might also consider presenting the unadjusted OR estimates | We made the figure with high resolution  We decided to the gender stratified description information in the background so that we not only talking about HCV results. As reviewer gave the liberty to decide authors  We revised table six.  We collapsed table 5 and 6 as advised and revised the text accordingly.  We also reported OR estimates as advised.  Please see table #6 |
